# Changing Use of Surgical Antibiotic Prophylaxis in Thika Hospital, Kenya: A Quality Improvement Intervention with an Interrupted Time Series Design

**DOI:** 10.1371/journal.pone.0078942

**Published:** 2013-11-11

**Authors:** Alexander M. Aiken, Anthony K. Wanyoro, Jonah Mwangi, Francis Juma, Isaac K. Mugoya, J. Anthony G Scott

**Affiliations:** 1 London School of Hygiene and Tropical Medicine, London, United Kingdom; 2 Kenya Medical Research Institute-Wellcome Trust Research Programme, Kilifi, Kenya; 3 Thika Level 5 Hospital, Thika, Kenya; 4 Kenyatta University, Nairobi, Kenya; 5 Ministry of Public Health and Sanitation, Nairobi, Kenya; 6 Nuffield Department of Clinical Medicine, John Radcliffe Hospital, Oxford University, United Kingdom; Kliniken der Stadt Köln gGmbH, Germany

## Abstract

**Introduction:**

In low-income countries, Surgical Site Infection (SSI) is a common form of hospital-acquired infection. Antibiotic prophylaxis is an effective method of preventing these infections, if given immediately before the start of surgery. Although several studies in Africa have compared pre-operative versus post-operative prophylaxis, there are no studies describing the implementation of policies to improve prescribing of surgical antibiotic prophylaxis in African hospitals.

**Methods:**

We conducted SSI surveillance at a typical Government hospital in Kenya over a 16 month period between August 2010 and December 2011, using standard definitions of SSI and the extent of contamination of surgical wounds. As an intervention, we developed a hospital policy that advised pre-operative antibiotic prophylaxis and discouraged extended post-operative antibiotics use. We measured process, outcome and balancing effects of this intervention in using an interrupted time series design.

**Results:**

From a starting point of near-exclusive post-operative antibiotic use, after policy introduction in February 2011 there was rapid adoption of the use of pre-operative antibiotic prophylaxis (60% of operations at 1 week; 98% at 6 weeks) and a substantial decrease in the use of post-operative antibiotics (40% of operations at 1 week; 10% at 6 weeks) in Clean and Clean-Contaminated surgery. There was no immediate step-change in risk of SSI, but overall, there appeared to be a moderate reduction in the risk of superficial SSI across all levels of wound contamination. There were marked reductions in the costs associated with antibiotic use, the number of intravenous injections performed and nursing time spent administering these.

**Conclusion:**

Implementation of a locally developed policy regarding surgical antibiotic prophylaxis is an achievable quality improvement target for hospitals in low-income countries, and can lead to substantial benefits for individual patients and the institution.

## Introduction

A World Health Organisation (WHO) systematic review in 2011 on hospital-acquired infections (HAI) highlighted the scarcity of studies from developing countries [1]. On the basis of limited information, Surgical Site Infections (SSIs) were identified as a particular problem: the risk in developing countries was *“strikingly higher than in equivalent surgical procedures in high income countries”*. A further WHO review confirmed the extremely high risk of SSI in African surgical patients [2].

Antibiotic prophylaxis in surgical patients is an effective means of reducing the risk of post-operative SSI [3] and a systematic review found that research studies conducted in sub-Saharan Africa supported this finding [4]. Antibiotic prophylaxis is most effective if given as a single intravenous injection approximately 30 minutes before the procedure starts [5], though the WHO Safe Surgery checklist indicates that it is acceptable to give prophylaxis between 0 and 60 minutes pre-operatively. No evidence supports the use of post-operative prophylactic antibiotics: scientific opinion is that these confer no benefit and should be avoided [6]. Nonetheless, many surgeons in both high- and low-income settings continue to use post-operative antibiotics: such prescribing increases costs and contributes to the selective pressure driving antibiotic resistance.

Achieving measureable and sustained improvements in the quality of healthcare is challenging in any setting, but in low-income countries examples of effectively introducing evidence-based practices are scanty [7]. Although the emergence of antimicrobial resistance could reverse much of the health gains achieved in Africa in the last half century [8], [9], there is a dearth of reports antibiotic stewardship interventions in Africa: a 2009 Cochrane Review of interventions to improve antibiotic prescribing for hospital inpatients found no reports from African countries [10].

We report our experience of developing and implementing a Surgical Antibiotic Prophylaxis (AP) policy as an intervention to change healthcare practitioners’ prescribing behaviour in a Government hospital in Kenya.

## Methods

### Study Design

During an 18-month period of SSI surveillance, we introduced a quality improvement intervention. We prospectively measured the effects of this intervention in terms of process, outcome and balancing measures, using various statistical methods to compare the time periods before and after this intervention.

### Study Setting

Thika Level 5 Hospital is a 300-bed Government Hospital in the town of Thika, approximately 50 km NE of Nairobi, Kenya. Six consultant surgeons and a rotating pool of 16–20 junior doctors and clinical officers (clinically-trained healthcare professionals) carry out approximately 300 elective and emergency operations monthly. We conducted SSI surveillance at Thika Hospital from August 2010 to December 2011 for all patients undergoing surgical procedures involving overnight admission to Thika Hospital. Patients remained in SSI surveillance for 30 days after all operations, encompassing both inpatient and outpatient periods. After discharge from hospital, we contacted patients by phone to determine the occurrence of post-discharge SSI. Further examination of the methodological approaches used for this surveillance is described elsewhere [11].

### SSI Surveillance

A team of hospital staff members (2 clinical officers and 4 support workers) conducted surveillance activities daily throughout the study period with on-site supervision by a clinical epidemiologist. Surgical wound class (Clean, Clean-Contaminated, Contaminated, Dirty) was assigned by the operating surgeon. No changes to surveillance methods or staff were made during this period. Surveillance staff, although not blinded to the introduction of the Antibiotic Prophylaxis policy, performed no role in its development or implementation.

### Outcome Measures

We diagnosed SSI in accordance with CDC-NHSN definitions [12] within the constraints of the diagnostic facilities available. All SSI diagnoses were based on clinical and radiological criteria - microbiological criteria were not used, although microbiology services at Thika Hospital were upgraded as part of the surveillance project. All SSI diagnoses were discussed with the relevant surgical team and an infectious diseases physician. For analytic purposes, we only considered the single anatomically deepest form of SSI (organ-space>deep> superficial) diagnosed in a patient during the 30 day surveillance period.

As process indicators, we analysed the proportion of patients undergoing surgical procedures who were documented to receive AP within 60 minutes of the start of the operation, and the proportion of patients prescribed post-operative antibiotics. Using weekly datapoints (26 pre- and 40 post-intervention), we used segmented regression analysis [13] to determine if there were significant step or slope changes in antibiotic prescribing behaviour associated with policy introduction.

As outcome measures, the risk of different forms of SSI within 30 days of the operation were recorded. We examined a time series plot of monthly datapoints (6 pre- and 9 post-intervention) for SSI risk. We looked for a step-change associated with initial policy introduction and for overall trends in risk over time by performing linear regression analyses for the periods before (August 2010–January 2011) and after (March–November 2011) policy introduction. In the absence of a clear pattern in the month–to –month variation in SSI risk, the overall risk in these periods was compared in a before-and-after format by calculating a risk ratio. Patients whose surgery was classified as “Contaminated” or “Dirty” were considered separately as work in Thika Hospital [11] and elsewhere [1] has shown that high levels of wound contamination to be strongly associated with increased SSI risk.

As balancing measures, we assessed financial costs and staff time requirements from the point of view of a hospital administrator. Costs were evaluated by comparing the average cost (per 100 operations) for purchasing and administering intravenous (iv) antibiotics based on usage in Thika Hospital during surveillance. The monetary values of drugs and other consumables were obtained from the awarded prices for medical products in Thika Hospital for 2011–12. We estimated the difference in nurse-hours based on an assumption of 10 minutes of nursing time per dose of intravenous medication being administered.

This study is reported in accordance with ORION guidelines [14]. Statistical analyses were done using STATA v12 except for the segmented regression analysis which was done using SAS v9.

### Ethics Statement

SSI Surveillance at Thika Hospital was approved by the Kenya Medical Research Institute National Ethics Review Committee. All patients gave written consent to participation in surveillance, which included contact by phone after discharge from hospital.

## Results

### Surveillance

We conducted SSI surveillance at Thika Hospital for all operations performed between the 16^th^ August 2010 and the 20^th^ November 2011 for patients who spent at least one post-operative night in hospital. We followed all patients for a 30-day post-operative period, with contact by telephone after discharge. A total of 3,343 patients were followed up in surveillance over this time (see table 1), with the most commonly performed procedures being Caesarean section (n = 2,594), laparotomy (n = 193), hysterectomy (n = 99) and hernia repair (n = 49).

**Table 1:** Surgical patients in SSI surveillance between August 2010 and November 2011.

Variable	Total number
**Patient characteristics**	
Age (years)	
≤14	84
15–39	2,936
40–65	289
65+	34
Sex	
Male	307
Female	3,036
**Operation characteristics**	
Type of surgery	
Caesarean section	2,594
General Surgery	327
Orthopaedic/Neurosurgery	125
Gynaecological	297
Surgeon grade*	
Consultant	525
Medical Officer	1,616
Medical Officer Intern	1,164
Registered Clinical Officer	38
Total operations followed up	3,343

* Medical Officer = junior doctor; Medical Officer Intern = junior doctor in first year after qualification; Registered Clinical Officer = vocationally-trained medical professional.

### Policy Development and Implementation

As our intervention, we developed an Antibiotic Prophylaxis (AP) policy for Thika Hospital over a series of multi-disciplinary seminars held between November 2010 and January 2011. This was based on reviewing African [15], [16], [17] and international [18] research papers and relevant national policy documents. The resulting AP policy recommended that for most routine operations (including Caesarean sections, hysterectomies and laparotomies), a combination of ampicillin (2g iv) and metronidazole (500 mg iv) should be given pre-operatively and that no antibiotics should be prescribed post-operatively. No restrictions were placed on use of antibiotics for post-operative prophylaxis, but it was agreed that there was no evidence to support this practice. The full policy is given in Appendix S1; this was endorsed by all consultant surgeons and anaesthetists working at Thika Hospital, the Medicines+Therapeutics Committee and the Medical Superintendant. As there was no regular supply of ampicillin from the Kenya Medical Supplies Agency (KEMSA) at this time, hospital pharmacists purchased this medicine locally.

Following extensive training of medical, nursing and theatre staff, this AP policy was implemented in Thika Hospital from the 7^th^ February 2011 onwards. Following an initial evaluation meeting, a patient information poster was used (Appendix S2) and personalised feedback was used to alert individual clinicians to substantial deviations from the practice of their colleagues. We estimate that a total of approximately 600 hours of staff time were used in meetings relating to development, implementation and evaluation of the AP policy.

### Results: Process Measures

**Figure 1:**
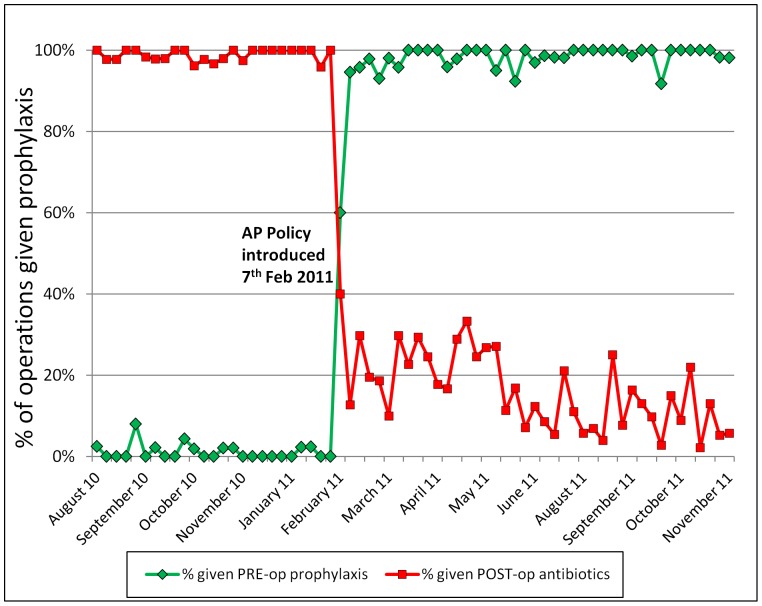
Weekly prescribing practices for surgical antibiotic prophylaxis.

In the pre-intervention phase of this study (prior to Feb 2011), less than 2% of patients were given pre-operative antibiotic prophylaxis – this was only used for patients having orthopaedic procedures involving implanted material – whilst over 99% of surgical patients were prescribed post-operative antibiotic regimes, typically a combination of penicillin, gentamicin and metronidazole given intravenously (iv) for three to five days, followed by a course of oral antibiotics. The implementation of the AP policy is illustrated in Figure 1, based on the weekly proportions of patients undergoing Clean or Clean-Contaminated surgery. Documented adherence to the AP policy (in terms of giving pre-operative prophylaxis) was 60% (27/45 operations) in week 1 and 98% (49/50) in week 6 after policy introduction. Use of post-operative prophylactic antibiotics in Clean and Clean-Contaminated surgery fell to 40% (18/45) in week 1 and 10% (5/50) in week 6 after policy introduction. Segmented regression analysis indicated that both the step change (p<0.0001) and the post-intervention downward trend (p = 0.001) were highly significant changes.

### Results: Outcome Measures

**Figure 2:**
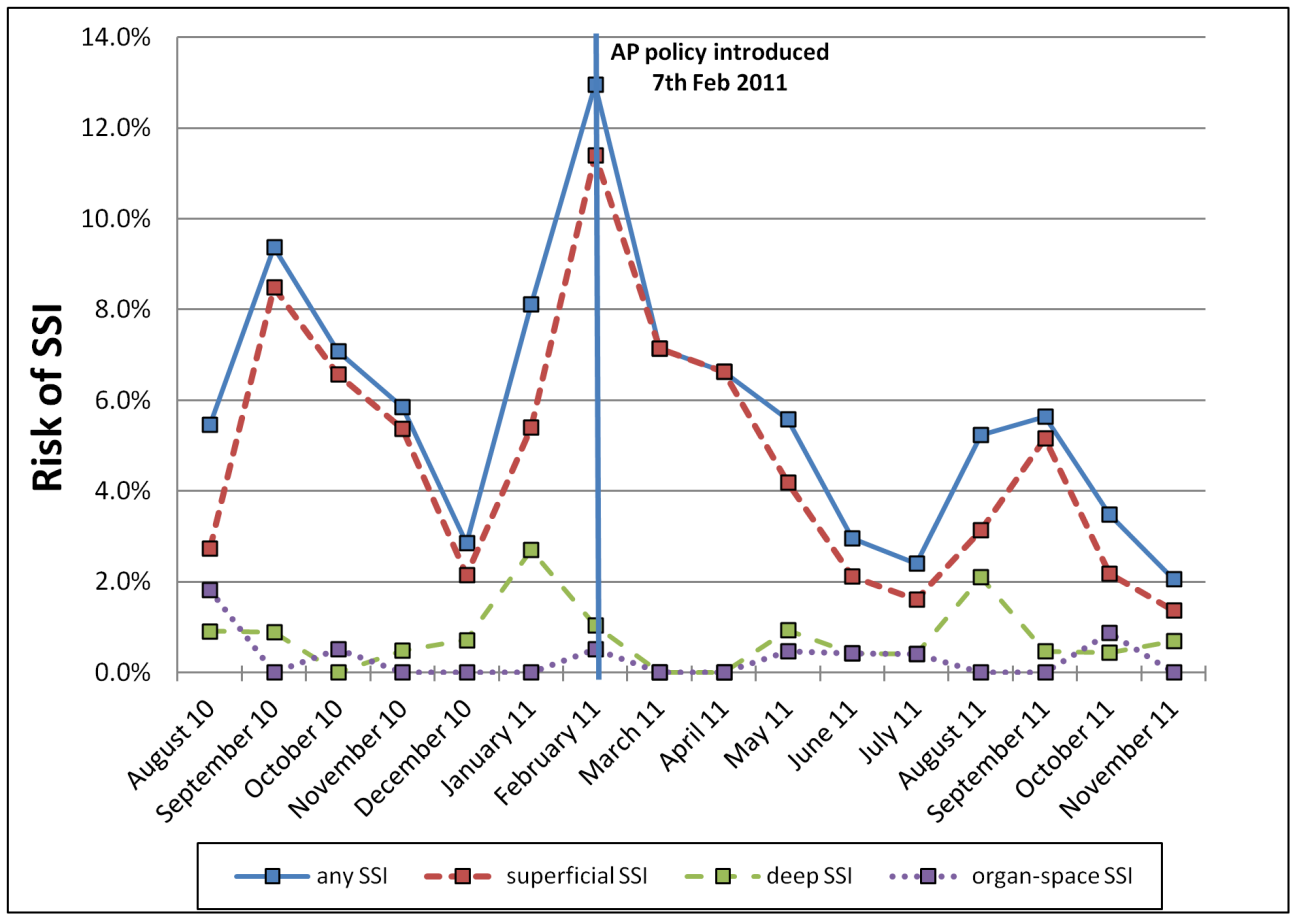
Monthly risk of SSI in Thika Hospital, 2010–11.

Over the surveillance period, the monthly risk of developing any form of SSI (superficial, deep or organ-space) ranged between 3 and 13% in patients whose surgery was classified as Clean or Clean-Contaminated (see Figure 2) with marked month-to-month variation. With linear regression, there was no clear evidence of a trend in risk prior to policy introduction (6 monthly datapoints; monthly change: −0.5%; 95% CI −2.5 to +1.4%, p = 0.49). After policy introduction there was some evidence of a downward trend in risk (9 monthly data points; monthly change: −0.7%; 95% CI −1.2 to −0.1%, p = 0.027). There was no indication of a step-change in SSI risk around the time of policy introduction.

Evaluation of the effects of the AP policy on SSI risk in a before-and-after comparison showed reduction in the risk of superficial SSI in both Clean/Clean-Contaminated surgery (RR 0.66; 95% CI 0.49–0.91; p = 0.01) and in Contaminated/Dirty surgery (RR 0.17; 95% CI 0.04–0.74; p = 0.005) – see Table 2. Changes in the risk of deep or organ-space SSI were found to be non-significant (p>0.1).

**Table 2:** Overall risk of SSI with and without use of pre-operative antibiotic prophylaxis.

Surgical Site Infections	Post-operative antibiotics only (%)	Pre-operative AP +/− post-operative antibiotics (%)	Risk Ratio (95% CI)	p-value^a^
**Clean and Clean-Contaminated surgery**				
number of operations	1,130 (100)	2,046 (100)		–
superficial	69 (6.1)	83 (4.1)	0.66 (0.49–0.91)	0.01
Deep	10 (0.9)	13 (0.6)	0.72 (0.32–1.63)	0.43
organ-space	3 (0.3)	6 (0.3)	1.10 (0.28–4.41)	0.89
total^b^	82 (7.3)	102 (5.0)		–
**Contaminated and Dirty surgery**				
number of operations	76 (100)	91 (100)		–
Superficial	10 (13.2)	2 (2.2)	0.17 (0.04–0.74)	0.006
Deep	6 (7.9)	13 (14.3)	1.81 (0.72–4.53)	0.20
organ-space	2 (2.6)	2 (2.2)	0.83 (0.12–5.79)	0.86
total^b^	18 (23.7)	17 (18.7)		–

a = p-value from χ^2^-test with 1 degree of freedom.

b = no RR calculated for all SSI combined as these represent diverse forms of infection.

### Results: Balancing Measures

The financial and time impacts associated with the introduction of the AP policy in Thika Hospital, based on recorded usage before and after AP policy introduction are given in Table 3. There was a net reduction in the costs for iv antibiotics and associated consumables of approximately $2.50/operation, and the number of iv injections administered and the nursing time spent performing these were both reduced by approximately 70%, leading to a saving of approximately 450 nurse-hours per month.

**Table 3:** Financial and other impacts associated with provision of surgical antibiotic prophylaxis, per 100 operations.

Item	Baseline August2010–Jan 2011	Intervention April2011–Nov 2011	Change associated with AP policy(% change from baseline)
**Costs (US$)**			
All iv antibiotic agents used †	394.21	293.14	−101.07 (b−26)
Consumables for iv administration	223.01	71.02	−151.99 (−68)
Total costs	617.22	364.16	−253.06 (−41)
**Other impacts**			
Doses of iv medications (number)	1221	367	−854 (−70)
Nursing time giving iv antibiotics (hrs)*	204	61	−143 (−70)

Exchange rate of Ksh85 =  US$1 used.

† based on documented prescriptions and number of doses administered in these time-periods.

* = based on an assumption of 10 mins nursing time/dose of iv antibiotics.

## Discussion

We believe this is the first report of the implementation of a quality improvement intervention in antibiotic use accompanied by process, outcome and balancing measures reported from sub-Saharan Africa. It is also the largest single-institution study of SSI risk in this region.

Our intervention, a locally-developed surgical antibiotic prophylaxis policy, rationalised clinicians’ prescribing, substantially reduced the use of intravenous antibiotics and saved both money and nursing time. Furthermore, there was some evidence of a modest reduction in risk of superficial SSI across all levels of wound contamination (RR 0.66 in Clean/Clean-Contaminated surgery; RR 0.17 in Contaminated/Dirty surgery).

We note that there was marked month-to-month variation in the overall risk of SSI during surveillance, principally due to changes in the recorded risk of superficial SSI. The highest overall risk of SSI was seen in the month of February 2011: this was both the month of the AP policy implementation and also the month that the annual intake of newly qualified junior doctors commenced work in Thika Hospital in 2011. This may have obscured the immediate effect of the changeover in AP use and may also account for the spike in SSI cases that occurred that month. Junior doctors (who perform the majority of uncomplicated Caesarean sections) rotated between hospital departments on a 3-monthly basis throughout the surveillance period; no other changes in staffing levels, equipment, facilities or operative techniques took place in Thika Hospital during this period.

### Limitations

Surgical Site Infections are inherently difficult to measure as they must be detected over a prolonged post-discharge period and largely rely on clinical judgement for diagnosis: these represent potential sources of classification error or bias. Although this study represents the largest report of SSI surveillance in a single institution in sub-Saharan Africa, it still remains relatively small in comparison to SSI surveillance studies in high-income settings. The study was almost certainly too small to detect changes in the risk of deep or organ-space SSI, which both occurred rarely. There are insufficient data points in this surveillance to discern cyclical patterns to SSI risk which could relate to hospital crowding or changes in levels of staff experience. Some circumstantial evidence, as described above, suggests that the latter could be important. This could be contributing bias to this study, though this would probably lead to underestimation of the effectiveness of the intervention as junior doctors were, on average, less experienced in the period after policy introduction.

Selection of antibiotics for use in the AP policy in Thika Hospital was difficult. Gentamicin was not used due to concerns about possible interactions with long-acting muscle relaxants. Co-amoxiclav might have been a better agent [16] as resistance to amoxicillin/ampicillin is widespread in Kenya [19] which might have lead to a more pronounced reduction in the risk of SSI, but at approximately five times greater cost for purchasing drugs.

### Barriers and Pathways to Change

Inappropriate antibiotic use is a multi-dimensional problem. What barriers existed to prevent this change in timing of antibiotic prophylaxis being implemented in Thika Hospital prior to 2011, when both research evidence and national guidelines supported such a change? Firstly, there was very limited awareness of national clinical policy documents, resulting in a marked policy-to-practice gap. Secondly, there was poor access to appropriate medicines for surgical prophylaxis – for example, although the Kenya Essential Medicines List describes ampicillin as “essential” for Government hospitals, in practice, this antibiotic was never received from official suppliers. Thirdly, amongst clinicians there was a marked concern about negative outcomes, especially regarding nosocomial infections, that hindered changes in antibiotic usage. The belief that prolonged post-operative antibiotic use mitigated the risk of HAI (in turn mainlyattributed to overcrowding and poor hygiene) was widespread. Finally, there was a lack of awareness amongst hospital management of the overall potential for cost-savings associated with such a policy change.

Why did this AP policy intervention succeed in changing local practice and sustaining this throughout the study period? Contributing factors to success may have been:

1. A strong evidence base and national infection control guidelines to support the policy.
2. “Buy-in” from senior clinicians and hospital management.
3. Multi-disciplinary and cross-departmental policy development.
4. Locally-relevant training materials for both staff and patients.
5. Extensive staff and patient sensitization prior to and after implementation.
6. Data collection for both process and outcome measures with timely feedback, including personalized feedback to persistent policy non-compliers.
7. Upgrade of SSI microbiology services as part of surveillance.
8. An appreciable saving of nurse-time and resources as a result of policy implementation.

Informal feedback from staff at Thika Hospital suggested that factors 5 and 8 were judged to have been most important, but all other factors played a contributing role. The change in practice with regard to use of surgical prophylaxis was reported to have been sustained as of August 2012, although no further data were collected.

## Conclusion

Antibiotic stewardship, evidence-based practice and infection control are all important issues in healthcare. Achieving lasting improvements in any one of these fields is difficult – but this intervention made an improvement in a typical Government hospital in Kenya in all three areas whilst also saving both nursing-time and resources. Many factors contributed to achieving successful implementation of policy – most importantly, local engagement with clinicians and support of the process of change.

Our report demonstrates that changing from the outdated practice of post-operative prophylaxis is an achievable quality improvement intervention for hospitals in low-income settings. The looming threat of widespread antibiotic resistance means that there is a pressing need to improve antimicrobial use in low-resource settings [20]. Better use of antibiotics for surgical prophylaxis is a “low-hanging fruit” [21] that is worth picking for antibiotic stewardship programmes in hospitals in sub-Saharan Africa.

## Supporting Information

Appendix S1 **Thika Hospital Surgical Antibiotic Prophylaxis Policy.**
(PDF)

Appendix S2 **Patient education poster in Kiswahili and English.**
(TIF)
