# Correction: Changing Use of Surgical Antibiotic Prophylaxis in Thika Hospital, Kenya: A Quality Improvement Intervention with an Interrupted Time Series Design

**DOI:** 10.1371/annotation/6506cc0b-2878-4cf8-b663-23a2f32a199a

**Published:** 2013-12-10

**Authors:** Alexander M. Aiken, Anthony K. Wanyoro, Jonah Mwangi, Francis Juma, Isaac K. Mugoya, J. Anthony G Scott

Professor Hajo Grundmann was not included in the author byline. Prof. Grundmann should be listed as the sixth author and affiliated with Department of Medical Microbiology, University of Groningen, University Medical Center Groningen, Groningen, the Netherlands. The contributions of this author are as follows: Conceived and designed the experiments, performed the experiments, analyzed the data, and wrote the manuscript.

In addition, there was an error in the Acknowledgments Statement. The Acknowledgments Statement should read: "The authors wish to thank the Thika Hospital staff and patients for their involvement in surveillance and the SSI Surveillance team for their diligent work on data collection and entry. We also wish to thank Andy Hall and Neal Alexander for their assistance with designing and conducting SSI surveillance and Peter Davey and Andrea Patton for advice on conducting an Interrupted Time Series analysis. " 

