# Supplementary material for: Changing Use of Surgical Antibiotic Prophylaxis in Thika Hospital, Kenya: A Quality Improvement Intervention with an Interrupted Time Series Design
Source: PLoS One. 2013 Nov 11;8(11):e78942. doi: 10.1371/journal.pone.0078942 (PMC3823974; doi:10.1371/journal.pone.0078942)
Supplement: Appendix S1 — Thika Hospital Surgical Antibiotic Prophylaxis Policy. (PDF) [file pone.0078942.s001.pdf]

<u>Thika L5 Hospital Surgical Antibiotic Prophylaxis (AP) Policy</u>

START: Patient in WARD/ CASUALTY

CONSULTANT / MO / MOI makes decision that patient is for operation

MOI /MO/CO inserts **<u>cannula</u>** for patient on morning of Operation (Elective Surgery) or as soon as decision to operate is made (Emergency Surgery).

This must be 18G size or larger.

MOI/ MO/CO writes a **<u>prescription</u>** for pre-operative AP on Treatment sheet

WARD NURSE checks the **<u>prescription</u>** has been written as part of Pre-Op Checklist and checks for drug allergies

WARD NURSE issues **<u>antibiotic prophylaxis (AP) drugs</u>** from ward stock. If unavailable on ward, NURSE requests **<u>AP drugs</u>** from PHARMACY on a named patient basis. If PHARMACY out of stock, patient or relatives must buy **<u>AP drugs</u>**

WARD NURSE records **<u>AP drugs</u>** dispensed in ward antibiotic book.

When MAIN/MATERNITY THEATRES ready to receive patient (ideally 30-45 mins prior to start of operation)

WARD NURSE transfers patient with **<u>cannula</u>**, **<u>prescription</u>** and **<u>AP drugs</u>** to THEATRE

RECEIVING NURSE in THEATRE only accepts patient if **<u>cannula, prescription</u>** and **<u>AP drugs</u>** are brought with the patient from the ward.

ANAESTHETIST confirms patient is fit for operation and the prescription for AP is appropriate

ANAESTHETIST / NURSE in THEATRE gives AP <u>aiming (ideally) for 30mins prior to skin incision</u> but can be given between 1 hour prior to incision (earliest) or with induction of anaesthesia (latest).

If it is an Emergency, <u>DO NOT DELAY</u> the OPERATION. AP can be given with induction of anaesthesia in emergency cases.

ANAESTHETIST and NURSES monitor for drug reactions to AP

If drug reaction to AP then NURSE to contact PHARMACY on ext 226 and fill in a **<u>Suspected Drug Reaction Form</u>**

THEATRE NURSE documents time and dose of pre-op AP in Patient notes (on Treatment sheet and Nursing Cardex)

SURGEON performs OPERATION

Patient is transferred back to WARD after operation

After OPERATION, no additional antibiotics are given on the ward <u>unless</u> TREATMENT is prescribed by SURGEON.

FINISH

Key:

WARD NURSE checks **<u>prescription</u>** has been written.

Each box describes a responsibility that a STAFF MEMBER has in the process of administering antibiotic prophylaxis (AP).

Pre-operative Antibiotic Prophylaxis for Surgical Patients in Thika Level 5 Hospital

Thika L5 Hospital Surgical Antibiotic Prophylaxis (AP) Policy

Operation Group	Antibiotics for PROPHYLAXIS	Dose Timing
Caesarean section: Elective OR Emergency*<br>*= Unless ruptured uterus / Prolonged ROM / Prolonged labour / other complication – see below	Ampicillin 2g<br>Flagyl 500mg	Single pre-op dose,<br>no post-operative antibiotics
Gynaecological Surgery – major procedures<br>(eg hysterectomy, oophorectomy, cystectomy)	Ampicillin 2g<br>Flagyl 500mg	Single pre-op dose,<br>no post-operative antibiotics
General Surgery "abdominal"<br>(eg laparotomy, appendisectomy (if no perforation), biliary tract surgery, colorectal surgery	Ampicillin 2g<br>Flagyl 500mg	Single pre-op dose,<br>no post-operative antibiotics
General Surgery "non-abdominal"<br>(eg hernia repair, mastectomy, thyroidectomy, burns grafting , fasciotomy)	Ampicillin 2g	Single pre-op dose,<br>no post-operative antibiotics
**CLEAN** Orthopaedic surgery and Neurosurgery<br>(eg ORIF, craniotomy, interlocking nail)	Ceftriaxone 2g	Single pre-op dose,<br>no post-operative antibiotics
EYE Surgery – Extensive EYE operations<br>(eg. Dacryocystorhinostomy (DCR), Enucleation of eye, repair of major eye trauma)	Ampicillin 2g<br>Flagyl 500mg	Single pre-op dose,<br>Give post-op Gentamycin + steroid eye drops
Local EYE surgery (cataract removal, glaucoma surgery)	Subconjunctival Gentamycin injection at end of op.	Give post-op Gentamycin +steroid eye drops
ENT Surgery – Extensive ENT operations<br>(eg parotidectomy, thyroglossal cyst removal)	Ampicillin 2g<br>Flagyl 500mg	Single pre-op dose,<br>no post-operative antibiotics
Local ENT operations (eg. Tonsillectomy, adenoidectomy)	No pre-operative AP	Post-op oral augmentin or azithromycin
Minor Gynaecology Procedures (on Ward or in theatre)<br>Eg. MVA, D+C.<br>(ESB and MacDonald stitch do not require AP)	Ampicillin 2g<br>Flagyl 500mg	Single dose before procedure,<br>Nil further treatment.
**ANY Contaminated or Dirty/Infected operation**<br>Including<br>• Surgical Toilet, Abscess drainage, arthrotomy for septic arthritis, traumatic wound closure, any gastro-intestinal perforation, amputation for gangrene.<br>• C/S with ruptured uterus/ Macerated Stillbirth/ PROM/Prolonged Labour.<br>• Any patient with an infection at the time of surgery (eg chorioamnionitis, infected wound, abscess).	Ampicillin 2g<br>Flagyl 500mg	Pre-operative PROPHYLAXIS AND then to received TREATMENT after operation as per clinicians prescription.
Patient with reported allergy to penicillin, for any surgery<br><br>*Note: there is a small risk of cross-allergy between Penicillins and Cephalosporins (approx 10% risk)	Omit Ampicillin from AP **if good history of allergy**. Can use Ceftriaxone* (2g) instead if necessary.	Single pre-op dose,<br>no post-operative antibiotics

Notes

- Patients should receive pre-operative Antibiotic Prophylaxis even if they are already on antibiotic treatment on the wards- these medicines are very unlikely to cause significant overdose.
- For children under the age of 10 years or adult patients less than 30kg, doses of antibiotics should be adjusted to the weight of the patient as follows: Ampicillin 50mg /kg, Metronidazole 7.5mg /kg.
- Repeat doses of AP should be given in operations lasting > 4 hours, or if major blood loss occurs.
- No additional post-operative treatment is required for immunosuppressed patients unless they have "Contaminated" or "Dirty" operation sites.
- Ampicillin is for use as Antibiotic Prophylaxis in Surgical patients. If an infection subsequently develops after surgery, different antibiotics should be prescribed for treatment of the infection as the organism(s) may be Ampicillin resistant. First line treatment for wound infections should be X-pen, Gentamicin, Flagyl.
- No oral antibiotics are needed on discharge if a patient had pre-operative AP and has no signs of wound infection at time of discharge. Prescribing further antibiotics wastes money and promotes drug resistance!
