# Supplementary figures and images for: Changing Use of Surgical Antibiotic Prophylaxis in Thika Hospital, Kenya: A Quality Improvement Intervention with an Interrupted Time Series Design
Source: PLoS One. 2013 Nov 11;8(11):e78942. doi: 10.1371/journal.pone.0078942 (PMC3823974; doi:10.1371/journal.pone.0078942)

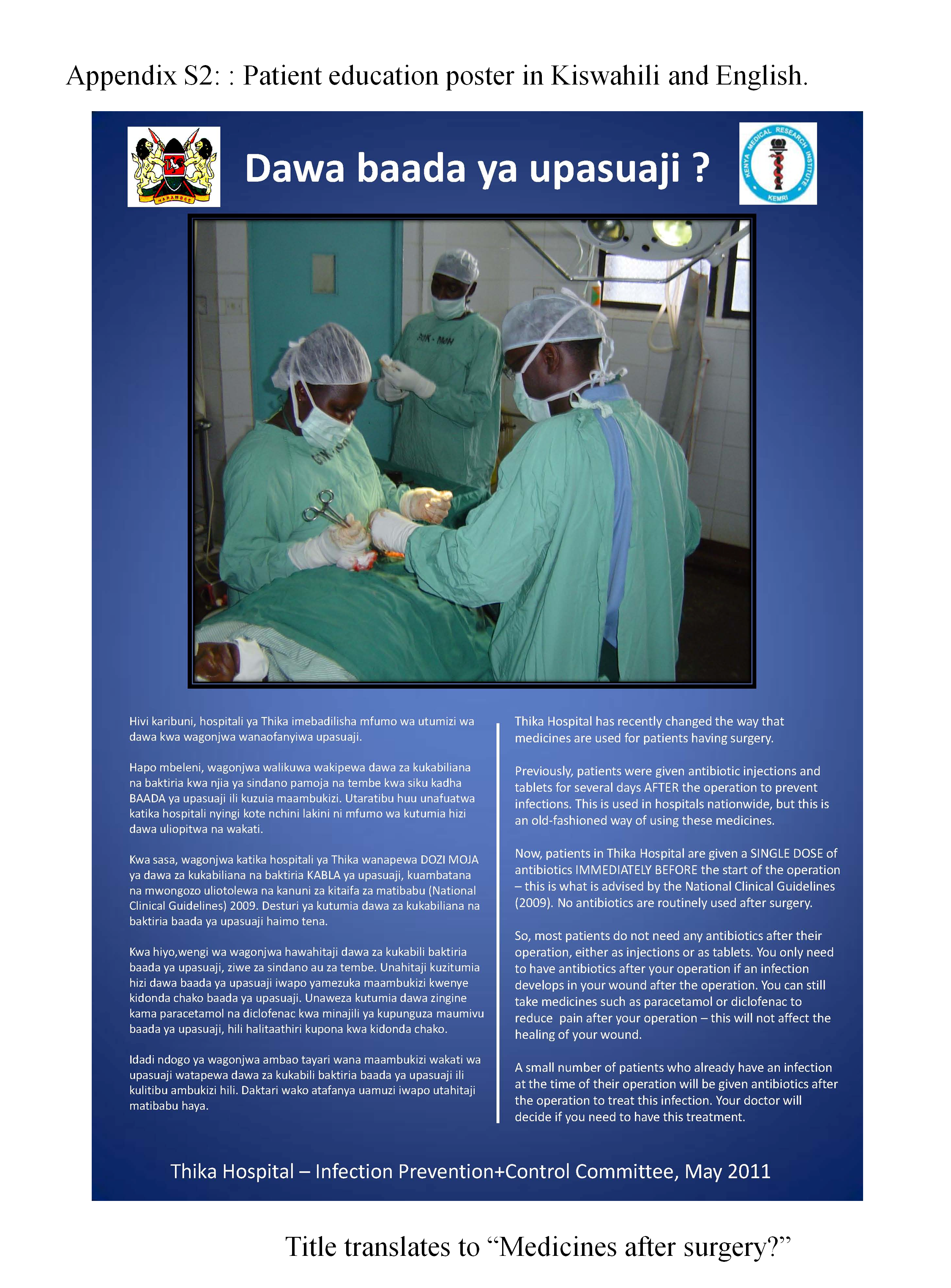

Supplement: Appendix S2 — Patient education poster in Kiswahili and English. (TIF) [file pone.0078942.s002.tif]
